# Intelligent *Eucommia ulmoides* Rubber/Ionomer Blends with Thermally Activated Shape Memory and Self-Healing Properties

**DOI:** 10.3390/polym15051182

**Published:** 2023-02-26

**Authors:** Qi Wang, Yutao Li, Jianbin Xiao, Lin Xia

**Affiliations:** Key Laboratory of Rubber-Plastics, Ministry of Education/Shandong Provincial Key Laboratory of Rubber-Plastics, School of Polymer Science and Engineering, Qingdao University of Science and Technology, Qingdao 266042, China

**Keywords:** shape memory, self-healing, *Eucommia ulmoides* rubber, ionomer

## Abstract

Intelligent *Eucommia ulmoides* rubber (EUR) and ionomer Surlyn resin (SR) blends were prepared and studied in this manuscript. This is the first paper to combine EUR with SR to prepare blends with both the shape memory effect and self-healing capability. The mechanical, curing, thermal, shape memory and self-healing properties were studied by a universal testing machine, differential scanning calorimetry (DSC) and dynamic mechanical analysis (DMA), respectively. Experimental results showed that the increase in ionomer content not only improved mechanical and shape memory properties but also endowed the compounds with excellent self-healing ability under the appropriate environmental conditions. Notably, the self-healing efficiency of the composites reached 87.41%, which is much higher than the efficiency of other covalent cross-linking composites. Therefore, these novel shape memory and self-healing blends can expand the use of natural *Eucommia ulmoides* rubber, such as in special medical devices, sensors and actuators.

## 1. Introduction

As a series of new functional materials, intelligent materials have greatly promoted the development of modern technology in the 21st century. Intelligent systems composed of these materials can sense and respond to external stimuli, mimicking the operation of living systems [1,2,3]. The emergence of information technology, high-end equipment manufacturing and the innovation of material research and technology drive the rapid development of advanced smart materials. Recently, in view of the unique properties of smart materials, such as the shape memory effect and damage–repair characteristics, related research has attracted extensive attention.

Shape memory materials are intelligent materials that can reversibly transform between their initial permanent shape and their temporary shape under specific environmental conditions (such as specified temperature, humidity, light, pH and magnetic and electric fields [4,5,6,7,8,9]). Compared to alloys and ceramics, the other two shape memory materials, polymers have the advantages of adjustable mechanical properties, good biocompatibility, light weights and low prices. Based on the above advantages, shape memory polymers (SMPs) can be widely used as important components in aerospace [10], biomedical devices [11,12,13,14] and sensing actuators [15,16].

Self-healing polymers (SHPs) are also responsive, intelligent materials that can repair damage and prolong the service life of products. Therefore, SHPs are of great significance in saving energy and reducing environmental pollution. According to the healing mechanism, SHPs can be divided into two categories: exogenous and endogenous. Exogenous SHPs usually need to incorporate external healing agents, the main forms of which include embedded microcapsules, filled hollow fibers and biomimetic, three-dimensional, microvascular networks [17,18,19]. Endogenous SHPs mostly rely on chemically reversible bonds to achieve a self-healing process. Some bonds, such as reversible ionic bonds [20,21], covalent bonds [22,23,24] and supramolecular chemical bonds, play an effective role in endogenous SHPs [25,26,27]. In addition, the effect of physical diffusion cannot be underestimated in SHPs [28,29].

In recent years, the excellent physical properties and fast responsiveness make intelligent, self-healing rubber materials promising candidates for flexible devices and long-life structural materials. To date, research on self-healing rubber materials mainly focuses on natural rubber (NR), butyl rubber (IIR), nitrile rubber (NBR) and other materials. For instance, Landro et al. investigated the self-healing behavior of epoxidized natural rubber (ENR) and cis-1, 4-polyisoprene (PISP) blends [30]. Heinrich et al. modified the bromine functional group of bromobutyl rubber (BIIR) into ionic imidazolium bromide groups to prepare highly elastic composites with extraordinary self-healing properties [31]. Carboxyl nitrile rubber (XNBR) was reported to be ionically cross-linked by zinc oxide (ZnO) to form ionic clusters, which exhibited a certain self-repairing ability and prolonged the life cycle of the rubber material [32].

However, there are still some problems to be solved in covalent cross-linking, self-healing rubber materials, such as low self-healing efficiency, high cost and complex preparation processes. Therefore, it is important to develop composite materials with excellent properties by simple, economic and efficient preparation methods. *Eucommia ulmoides* rubber (EUR) is mainly composed of trans-1, 4-polyisoprene, which can be used as a shape memory material with a low cross-linking density [33,34]. EUR exhibits excellent shape memory properties, which enables the material to recover its original shape when subjected to external forces such as stretching, compression, etc. There are relatively few research papers on the shape memory properties and self-healing properties of Eucommia rubber. Yue et al. modified Eucommia ulmoides rubber by the epoxidation method and prepared a composite with both shape memory and self-healing properties with a tensile strength of ~12.1 MPa and elongation of ~876% [35].

Here, we manufactured EUR- and SR-based blends with a moderately cross-linking approach. Surlyn resin (SR) is an ethylene–methacrylic acid copolymer thermoplastic resin whose internal ionic bonds give it unique properties and potential applications for self-healing [36,37,38,39,40,41]. Semi-crystalline and lightly cross-linked EUR and SR enable blends to complete shape memory processes with proper programming procedures while electrostatic interactions of ionomers provide the prerequisite for self-healing. The blends with a EUR/SR blending ratio of 10/90 showed an R_f_ of 96.46% and an R_r_ of 82.47%, respectively, and EUR/SR blends with a blending ratio of 30/70 achieved the highest self-healing efficiency of 87.41%, which was much higher than the self-healing efficiency of ~40% at the same blending ratio (SR/PCO = 70/30) in similar Surlyn 9520 ionomer and polycyclooctene systems [42]. Therefore, this work provides a convenient and facile approach for manufacturing rubber-based composites with high shape memory and high self-healing properties. EUR/SR blends have potential applications in medical devices and various sensor fields.

## 2. Experimental Procedure

Herein, we demonstrate, for the first time, the fabrication of a composite with excellent mechanical shape memory and self-healing properties by using EUR and SR as the matrix via a simple blending method. The double cross-linking network constructed by the covalent cross-linking of the EUR component and the ion cross-linking of the SR component gave the composite the dual effect of heat-induced shape memory and self-healing. Significantly, the self-healing efficiency of the composites reached 87.41%, which is difficult for other covalent cross-linking materials to achieve.

### 2.1. Materials

**Figure 1:**
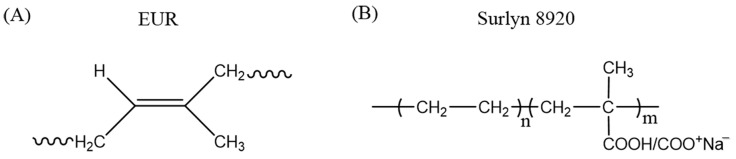
Chemical structure of (**A**) EUR and (**B**) SR.

The bark of *Eucommia ulmoides* was crushed and dissolved in the organic solvent chloroform, the dissolved EUR was precipitated using freezing ice water and then the gum was filtered to obtain solid EUR. The lyophilized resin 8920 Na-ionomer contained methacrylic acid groups (5.4 mol%) and MA groups, sixty percent of which were neutralized by sodium (DuPont Co., Ltd., Wilmington, DE, USA). The chemical structure of EUR and SR is shown in Figure 1. Dicumyl peroxide (DCP, purity 96%) was purchased from Aladdin Co., Ltd. (Shanghai, China). Other reagents were obtained from commercial sources and used without further purification.

### 2.2. Preparation of the Blends

EUR and SR blends were prepared by a high-temperature, open mill with a temperature of 90 °C for approximately 10 min using a standard mixing sequence. After the melting and mixing process, these composites were pressed into plates at 160 °C with a thickness of 2 ± 0.1 mm. The compound formulations are shown in Table 1.

### 2.3. Curing Characteristics

The curing characteristics of the EUR/SR blends were monitored by a Monsanto oscillating disc rheometer (MDR-2000, GOTECH TESTING MACHINES Inc., Taichung, Taiwan) at 160 °C, according to ASTM D-2084-11, and torque was recorded over time. The scorch time T_10_, optimum cure time T_90_, minimum torque and maximum torque were determined from curing graphs.

### 2.4. Mechanical Characterization

Vulcanized slabs were dumbbell-shaped by die-cutting according to ASTM Die C. The mechanical tests were conducted following the ASTM D-412-16 and ASTM D-624-00 standard procedures using a tensile testing machine (AI-7000S, High-Speed Rail Co., Ltd., Taiwan). For each set of samples, five splines were tested and the average value was used as the experimental result. In addition, according to ASTM D2240-2015, the hardness of the composites was determined using a hardness tester (LX-A, Shanghai Liuling Instrument Factory, Shanghai, China).

### 2.5. Differential Scanning Calorimetry (DSC)

DSC measurements were carried out on a DSC-Q20 (TA Instruments, New Castle, DE, USA) under a nitrogen atmosphere over the temperature range from −50 to 120 °C at a heating speed of 10 °C min^−1^. Samples were maintained at 120 °C for 3 min to eliminate their thermal histories before cooling. The exothermic curves of heat flow as a function of time were recorded to calculate the degree of crystallinity (X_c_) for each portion of the composites using Equation (1):

(1) $$Xc=\frac{\Delta H_{m}}{\Delta H_{m}^{*}}\times 100\%$$

where ΔH_m_ and ΔH_m_* are the melting enthalpy of the polymer and its theoretical melting enthalpy (ca. 186.8 J∙g^−1^ for EUR), respectively. ΔH_m_ in Surlyn resin is calculated by the mass fraction of the target component.

### 2.6. Shape Memory Effect

A TA Instruments DMA Q800 with the initial clamp gap set to 5.0~10.0 mm was used to analyze the shape memory properties of the composites. The specifications of the test sample and the test procedure for the shape memory effect were the same as the works we reported earlier [43,44]. First, the sample was maintained isothermally at 120 °C to melt the crystalline regions completely. Then, a load of 0.02 MPa was applied, after which the sample was cooled to −20 °C to freeze the crystalline domain completely. Then, the load was removed, and the sample was reheated to 120 °C and isothermally maintained for 15 min. The critical parameters for shape memory effect (SME) characterization, shape fixity ratio (R_f_) and shape recovery ratio (R_r_) can be quantified as follows:

(2) $$R_{f}\left(0\to 1\right)=\frac{\varepsilon_{1}-\varepsilon_{0}}{\varepsilon_{1,\mathrm{load}}-\varepsilon_{0}}\times 100\%$$

(3) $$R_{r}\left(1\to 0\right)=\frac{\varepsilon_{1}-\varepsilon_{0,\mathrm{rec}}}{\varepsilon_{1}-\varepsilon_{0}}\times 100\%$$

where ε_0_ is the initial strain, ε_1,load_ represents the maximum strain under the load, ε_1_ is the strain after cooling and load removal, and ε_0,rec_ is the recovered strain.

### 2.7. Self-Healing Effect

To perform the self-healing test, the standard dumbbell-type tensile splines were first cut into two independent parts from the middle with a blade, the fracture surfaces were spliced and the healing process started at 120 °C and 10 MPa pressure. Finally, the specimens were left to stand at room temperature for 5 days. The healing efficiency was quantified by comparing the tensile strengths of the healed sample and the original sample by stretching to fracture at a cross-head velocity of 500 mm·min^−1^ at 25 °C, as shown in Equation (4). For each set of samples, three splines were tested and the average value was used as the tensile strength. In addition, the self-healing cracks were sprayed with gold and placed on the aluminum base with conductive tape to observe the surface morphology under a scanning electron microscope (SEM) (JEOL JSM-6700F, Tokyo, Japan).

(4) $$\mathrm{Healing}\;\mathrm{efficiency}=\frac{{\sigma b}^{\mathrm{healed}}}{{\sigma b}^{\mathrm{pristine}}}\times 100\%$$

## 3. Results and Discussion

### 3.1. Curing and Mechanical Properties

Table 2 shows the curing characteristics of the composites with different blend ratios. The minimum torque and maximum torque during the curing process were marked as *M_L_* and *M_H_*, respectively, while values of *M_H_-M_L_* usually indicated the degree of cross-linking of the composites, and all of the above parameters showed a decrease with the increase in the Surlyn resin content. The scorch time (T_10_) and the optimum cure time (T_90_) of these composites both increased significantly with increasing Surlyn resin content. In particular, T_90_ increased from 21.85 min to 30.50 min, which implies that the increase in the amount of starch resin slowed the vulcanization efficiency and improved the processing safety of the composites. The reason for the increase in T_10_ and T_90_ was that the reduction in *EUR* components in these composites prolonged the scorch time and curing time with the same amount of curing agent. The value of *M_H_-M_L_* reflected a decrease in the total cross-linking density of the material as the EUR/SR resin blending ratio decreased, which could be ascribed to the poor cross-linking effect of DCP for the EUR/SR system in which DCP tended to be distributed in the EUR phase.

To clarify the relationship between the EUR/SR blend ratio and the mechanical properties of the composites, some important mechanical parameters were measured and are shown in Table 3. Interestingly, the tensile strength, 100% modulus, elongation at break, tear strength and hardness of the composites all increased with the decreasing EUR/SR blend ratio. The main reason for this phenomenon was that SR exhibited a plastic behavior, increasing the tensile strength, 100% modulus, elongation at break, tear strength and hardness of the composites. On the other hand, DCP had a different cross-linking effect for the EUR and SR components such that when the composites were subjected to longitudinal tensile force, the EUR phase with a higher cross-linking density gave the system a higher tensile strength, and the SR phase with a relatively lower cross-linking density afforded a larger elongation at break. When the EUR/SR blend ratio was 10/90, the mechanical properties of the composites were the best, exhibiting a tensile strength of 16.59 MPa, 100% modulus of 14.46 MPa and tear strength of 110.21 KN·m^−1^.

### 3.2. DSC Analysis

EUR crystallizes easily because of its regular trans-structure of macromolecular chains. The crystal zone plays an important role in composites. The crystal zone can increase the mechanical properties of the composites. On the other hand, it can be used as a recovery phase and healing phase to improve the shape memory and self-healing properties of composites separately. Therefore, it is critical to investigate the effect of the blend ratio on the crystallization of the composites.

**Figure 2:**
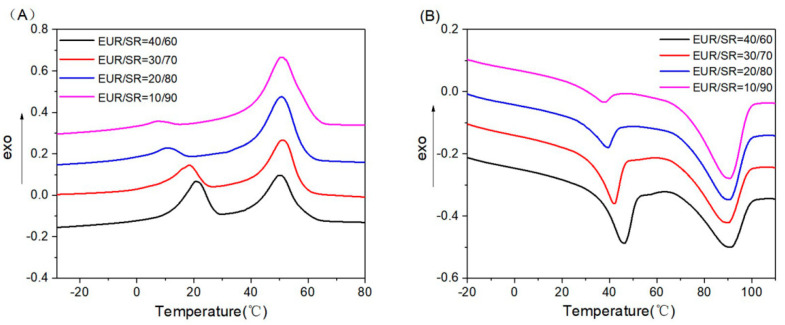
Differential Scanning Calorimeter (DSC) curves of the EUR/SR blends with different blend ratios: (**A**) cooling curves and (**B**) heating curves.

Both the thermal properties and transition temperatures of these blends were measured by DSC (Figure 2). The crystalline degree and melting range in Table 4 were obtained from DSC curves. As shown in Figure 2 and Table 4, the melting point of the EUR component decreased from 45.36 °C to 36.54 °C with the reduction in the EUR content. The X_c_ of the blends also decreased from 21.54% to 17.51%, since the addition of DCP peroxide caused a cross-linking reaction of the EUR phase and destroyed the crystallization. Meanwhile, the peroxide DCP also had a certain sulfurization cross-linking effect on the SR component. Therefore, the melting point of the SR component increased from 88.74 °C to 90.53 °C, and the crystallinity of the SR component increased from 12.00% to 12.77% with increasing SR content. The thermal properties of the two components in the blends provided data support for the study of the shape memory and self-healing properties of these blends.

### 3.3. Shape Memory Effect Analysis

**Figure 3:**
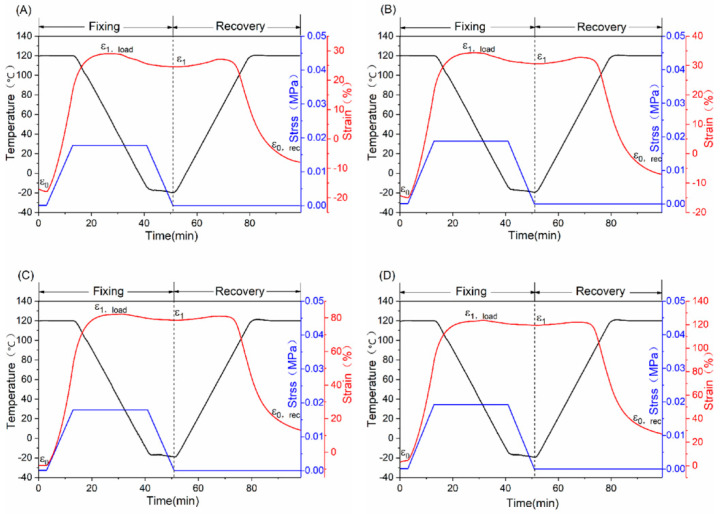
Shape memory properties of the EUR/SR blends with different blend ratios: (**A**) 40/60; (**B**) 30/70; (**C**) 20/80; (**D**) 10/90.

A dynamic mechanical analyzer (DMA) was used to characterize the thermo-induced shape memory properties of EUR/SR blends in this work. We characterized the shape memory properties of the blends with different blend ratios under different heat conditions. Figure 3 shows the shape memory properties of the EUR/SR blends with different blend ratios. According to the procedure set in advance, the crystalline regions of the EUR and SR, acting as reversible domains in the blends, were first melted when the blends were heated. Meanwhile, the cross-linking network acted as a fixed domain to restrict the plastic slippage of the molecular chains. As a result, the blends were reversibly transformed between initial permanent and temporary shapes during the heating-loading-cooling-unloading-reheating process. In the shape fixing and restoring process, we could accurately calculate the shape fixity ratio (R_f_) and the shape recovery ratio (R_r_) of the blends using the DMA test. Figure 3 and Table 5 show that the maximum strain difference (ε_1,load_ − ε_0_) under the load increased from ~50% to ~120% when the blend ratio of EUR/SR changed from 40/60 to 10/90, which confirmed the increasing plasticity and decreasing cross-linking density of the whole system and leads to the enhancement in the ductility of composites. The R_f_ and R_r_ of the blends showed a gradually increasing trend with increasing SR content. The value of the former increased from 90.10% to 96.46%, and the latter increased from 75.78% to 82.47%.

**Figure 4:**
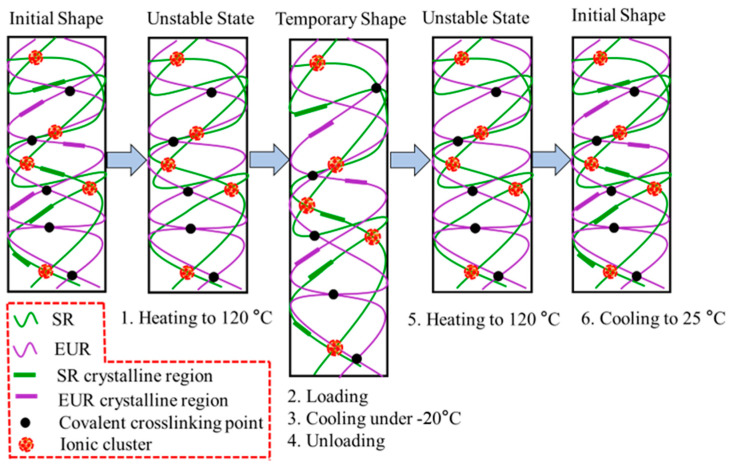
Scheme diagrams for shape memory effect of EUR/SR blends.

**Figure 5:**
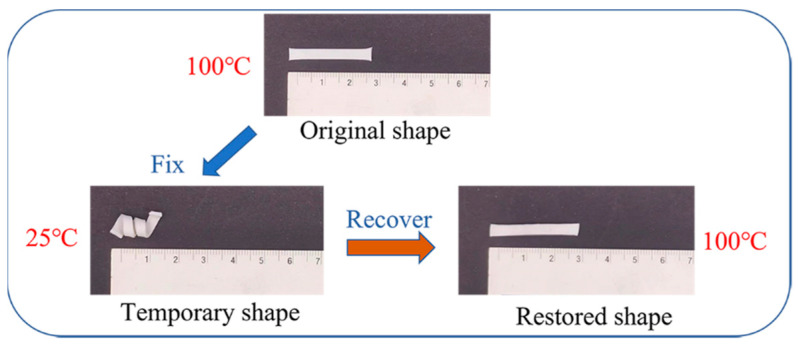
Visual photograph of shape memory behavior for EUR/SR blends.

A schematic diagram is proposed to explain the shape memory behavior of EUR/SR blends. In Figure 4, the random lines and the regular rectangles represent amorphous molecular chains and crystalline regions, respectively. The black spots represent the covalent cross-linking points in the composites, and the pink dots represent the self-aggregation of Na+ ion pairs, which formed ionic clusters and restricted the mobility of SR molecular chains in the blends. Figure 4 shows the whole shape memory process of the blends. When the temperature was kept constant at 120 °C, all of the crystalline regions in the composites were melted. At this point, the material was deformed by applying an external load. After a subsequent cooling and unloading process, the crystalline phase fixed the temporary shape as a stationary domain. When heated again, the reversible crystalline phase disappeared, and the cross-linking network of covalent and ionic bonds provided contraction, returning it to its original shape. Therefore, when the EUR/SR blending ratio in the composites decreased, the R_f_ continued to increase and finally approached 100% because the increasing crystallinity of the dominant SR component improved the fixing ability of the temporary shape. However, R_r_ only exhibited a slight uptrend. Although DCP had a certain cross-linking effect on both components, especially increasing the entropy elasticity of the EUR-component cross-linking network, the EUR content was constantly reducing, which weakened the role of its cross-linking network. Based on the above factors, the increase in R_r_ was not significant. In order to study the shape memory behavior of EUR/SR blends, a sample with a 10/90 blending ratio was chosen and subjected to visual observation at room temperature. As shown in Figure 5, a long strip of the sample was heated to 100 °C and then manually stretched until it was wound around a cylinder with a diameter of roughly 0.5 cm. Finally, this spiral sample was left at a temperature of 25 °C. During the recovery process, the spiral sample was able to unwind into a flat strip and recover approximately to its initial shape. Thus, the shape memory behavior of these blends of composites can be achieved through high-temperature (100 °C) and low-temperature (25 °C) processes.

### 3.4. Self-Healing Analysis

**Figure 6:**
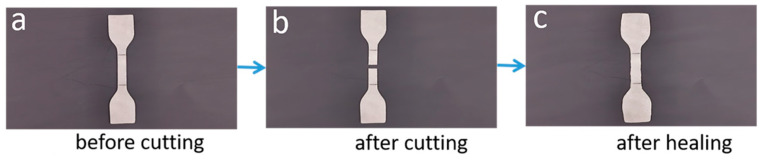
Representative photographs of the healing process of EUR/SR blends.

**Figure 7:**
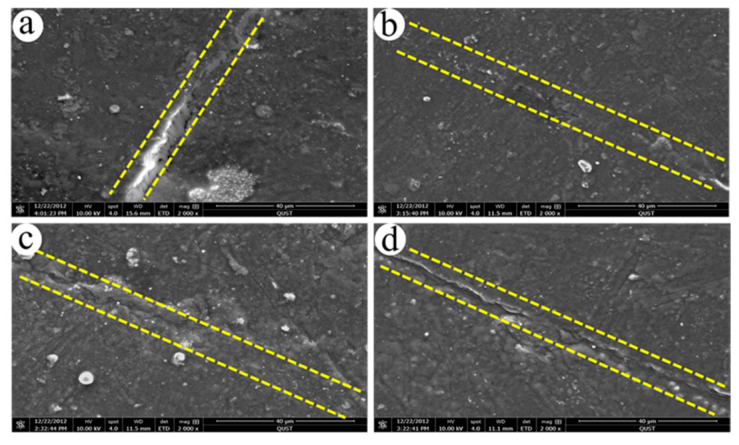
SEM images of EUR/SR blends after self-healing with different blend ratios: (**a**) 40/60, (**b**) 30/70, (**c**) 20/80, (**d**) 10/90, all healed at 120 °C for 30 min.

The images of the specimens before and after self-healing are shown in Figure 6. There was no obvious difference between the healed specimens and the original specimens. We further observed the microscopic morphology of the self-healing regions in Figure 7. The appearance of the healed cracks could be clearly observed. In particular, the healed section in Figure 7b was relatively smooth compared with Figure 7a,c,d. The tensile strength was further tested to measure the healing efficiency of the composites, as shown in Table 6. As expected, the failure of these specimens still occurred at the healed joint in the tensile experiment. These blends showed excellent mechanical properties after healing. When the EUR/SR ratio was 30/70, the tensile strength of the blends achieved 12.64 MPa, and the healing efficiency reached 87.41%. The mechanical behaviors of the composites were related to the microstructure of the composites. There were crystalline structures, random structures and cross-linking structures of EUR and SR components in the composites. The mechanical properties of the composites depended on the density of the cross-linking networks, the size and content of the crystalline phase regions and the diffusion entanglement of molecular chains. The mechanical properties of the blends after healing were mainly related to the free diffusion of molecular chains and ionic interactions at the cross-section in the blends. Meanwhile, the density of the cross-linking network in the blends limited the movement of molecular chains and chain segments, thus affecting the diffusion of molecular chains. Therefore, we believe that the internal microstructure of the blends, including the cross-linking network, crystal structure and diffusion entanglement of molecular chains, determined the properties of the blends.

**Figure 8:**
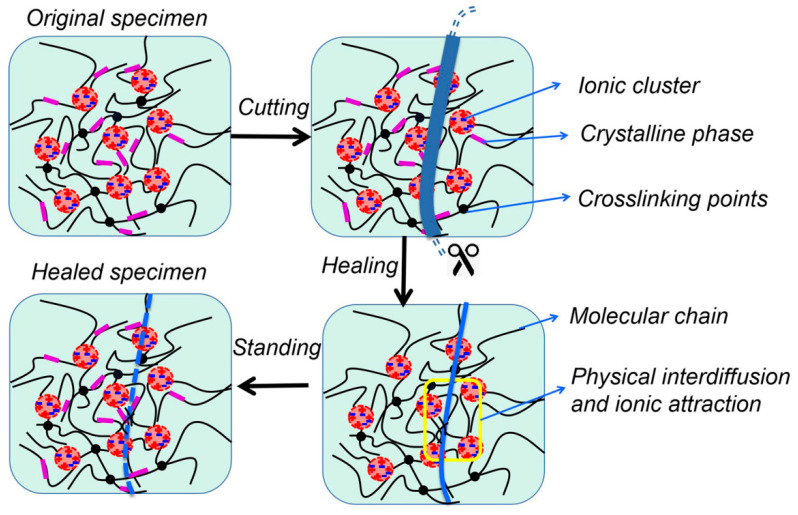
Scheme diagrams for self-healing behaviors of EUR/SR blends.

We also propose a microscopic schematic diagram to vividly explain the self-healing behavior of EUR/SR blends in Figure 8. In the schematic diagram, the black lines represent molecular chains of polymers, black dots represent cross-linking points, red aggregation balls represent ion cluster structures and purple rectangles represent crystallization regions in the composites. First, the specimens were cut, and the fresh outer surface was exposed. Then, the sections were spliced together. By heating, the molecular chains on the surface of the cross section diffused freely. Meanwhile, the electrostatic mutual attraction between the ion clusters in the ionomer component also accelerated the diffusion of molecular chains. In the melting state, polymer chains diffused and entangled each other because of the existence of intermolecular, electrostatic attraction in the ion zone of the fractured cross section in the flow process, thereby repairing the crack. Furthermore, the crack strength was enhanced through subsequent crystallization at room temperature.

## 4. Conclusions

We prepared EUR/SR blends with different blending ratios and demonstrated their excellent properties. The cross-linked network constructed by covalent and ionic bonds endowed the composites with excellent mechanical properties, sensitive thermal stimulus response shape memory and self-healing properties. Notably, the self-healing efficiency of the composites was found to be 87.41%, which is much higher than the efficiency of other covalent cross-linking composites. Therefore, these novel EUR/SR composites might expand the use of natural *Eucommia ulmoides* rubber, such as in special medical devices, sensors and actuators.

## Figures and Tables

**Table 1:** Formulations of EUR/SR Blends.

Formulation	A	B	C	D
EUR	40	30	20	10
Surlyn resin	60	70	80	90
ENR	3	3	3	3
DCP	1	1	1	1
Antioxidant MB	1.5	1.5	1.5	1.5

**Table 2:** Curing Characteristics of EUR/SR Blends.

Properties	Blends with Different Ratios of EUR/SR			
	A	B	C	D
T_10_ (min)	1.85	2.07	2.36	2.80
T_90_ (min)	21.85	24.76	28.18	30.50
M_L_ (dN·m)	0.6	0.5	0.5	0.4
M_H_ (dN·m)	2.5	2.2	1.8	1.5
M_H_-M_L_ (dN·m)	1.9	1.7	1.3	1.1

**Table 3:** Mechanical Properties of EUR/SR Blends.

Properties	Blends with Different Ratios of EUR/SR			
	A	B	C	D
Tensile strength (MPa)	13.82 (±0.50)	14.46 (±0.35)	14.68 (±0.42)	16.59 (±0.41)
100% modulus (MPa)	12.22 (±0.50)	12.78 (±0.35)	13.20 (±0.41)	14.46 (±41)
Elongation at break (%)	175 (±21)	183 (±11)	195 (±14)	206 (±15)
Tear strength (KN·m^−1^)	91.04 (±0.68)	94.33 (±0.48)	102.55 (±0.51)	110.21 (±0.51)
Hardness (Shore A)	92	93	93	94

**Table 4:** Crystallinity of EUR/SR Blends.

Properties	Blends with Different Ratios of EUR/SR			
	A	B	C	D
T_m (EUR)_ (°C)	45.36	41.69	38.56	36.54
T_m (SR)_ (°C)	88.74	89.55	89.69	90.53
X_c (EUR)_ (%)	21.54	20.09	18.90	17.51
X_c (SR)_ (%)	12.00	12.34	12.42	12.77

**Table 5:** Shape Memory Properties of EUR/SR Blends.

Properties	Blends with Different Ratios of EUR/SR			
	A	B	C	D
R_f_ (%)	90.10	92.16	95.78	96.46
R_r_ (%)	75.78	78.87	78.97	82.47
ε_1,load_ − ε_0_ (%)	~50	~55	~85	~120

**Table 6:** Mechanical Properties of Healed EUR/SR Blends.

Properties	Blends with Different Ratios of EUR/SR			
	A	B	C	D
Tensile strength (MPa)	6.12 (±0.93)	12.64 (±0.76)	12.19 (±0.72)	10.95 (±0.65)
Elongation at break (%)	1.12 (±0.16)	18.17 (±2.31)	13.69 (±1.74)	2.24 (±0.56)
Healing efficiency (%)	44.28	87.41	83.04	63.83

## Data Availability

All the data used to support the findings of this study are included within the article.
